# Genetically engineered pigs as models for human disease

**DOI:** 10.1242/dmm.030783

**Published:** 2018-01-01

**Authors:** Carolin Perleberg, Alexander Kind, Angelika Schnieke

**Affiliations:** Chair of Livestock Biotechnology, School of Life Sciences, Technische Universität München, 85354 Freising, Germany

**Keywords:** Disease models, Genetic modification, Pig, Swine

## Abstract

Genetically modified animals are vital for gaining a proper understanding of disease mechanisms. Mice have long been the mainstay of basic research into a wide variety of diseases but are not always the most suitable means of translating basic knowledge into clinical application. The shortcomings of rodent preclinical studies are widely recognised, and regulatory agencies around the world now require preclinical trial data from nonrodent species. Pigs are well suited to biomedical research, sharing many similarities with humans, including body size, anatomical features, physiology and pathophysiology, and they already play an important role in translational studies. This role is set to increase as advanced genetic techniques simplify the generation of pigs with precisely tailored modifications designed to replicate lesions responsible for human disease. This article provides an overview of the most promising and clinically relevant genetically modified porcine models of human disease for translational biomedical research, including cardiovascular diseases, cancers, diabetes mellitus, Alzheimer's disease, cystic fibrosis and Duchenne muscular dystrophy. We briefly summarise the technologies involved and consider the future impact of recent technical advances.

## Abstract (precis)

**Summary:** An overview of porcine models of human disease, including cardiovascular diseases, cancers, diabetes mellitus, Alzheimer's disease, cystic fibrosis and Duchenne muscular dystrophy. We summarise the technologies involved and potential future impact of recent technical advances.

## Introduction

New therapies and diagnostic methods are required for many human diseases. There are, however, no *in vitro* systems capable of modelling human whole-body pathophysiology; as such, disease research still relies on animals. Work with laboratory animals is carefully controlled, and workers in the field have a duty to ‘replace, reduce and refine’ their use whenever possible [see Article 47 of Directive 2010/63/EU (http://eur-lex.europa.eu/legal-content/EN/TXT/PDF/?uri=CELEX:32010L0063&from=EN) and www.nc3rs.org.uk]. It is thus important to ensure that all data gained are valuable and relevant to the disease studied. This is best achieved with well-defined animal models that replicate relevant aspects of human pathology as closely as possible.

Mice are now the most intensively studied and widely used mammalian species in biomedical research, mainly because they are convenient and cheap to house, and methods for their genetic modification are well advanced (Chu et al., 2016; Skarnes et al., 2011). Mouse studies have provided a wealth of information on the molecular basis of human disease and have enabled a host of proof-of-principle studies. Mice, however, do not always accurately model human disease pathology, reducing their predictive value for preclinical studies (Mak et al., 2014). Many new drugs fail in clinical trials because preclinical studies fail to predict safety and effectiveness in human patients (Justice and Dhillon, 2016; Ledford, 2011). Nonrodent species can provide additional information and improve the predictive value of preclinical studies (Bähr and Wolf, 2012).

Pigs share several key similarities with humans in terms of their body size, anatomical features, physiology, pathophysiological responses and diet, and are used to develop and refine biomedical procedures and medical equipment (Heinritz et al., 2013; Kararli, 1995; Schubert et al., 2016). Their use in biomedical research is aided by several practical factors, such as their favourable breeding characteristics. Pigs mature relatively quickly for a large species (6-7 months), have a short gestation period (∼114 days) and produce large litters (∼10 piglets per litter), depending on the breed (Sachs, 1994). Centuries of pig domestication have established suitable housing conditions, including specific pathogen-free conditions, which require only minor adaptation for research. Furthermore, as food animals, there is wide public acceptance of their humane use, which is not the case for other nonrodent species, such as primates.

The extension of genetic modification technology to pigs has greatly increased their value to biomedicine, motivating efforts to develop porcine models that replicate human disease, and so ‘bridge the gap between bench and bedside’. This review outlines the techniques used and describes the most promising and relevant porcine models of human disease (summarised in Table 1). This is, however, not a comprehensive account of all genetically modified pigs, and some reports of nonviable animals or animals with no relevant phenotype have been omitted. Our aim is to highlight the importance of porcine disease models for translational biomedical research, and to indicate those porcine models that recapitulate human disease pathology most accurately and those with the greatest potential.

**Table 1.**
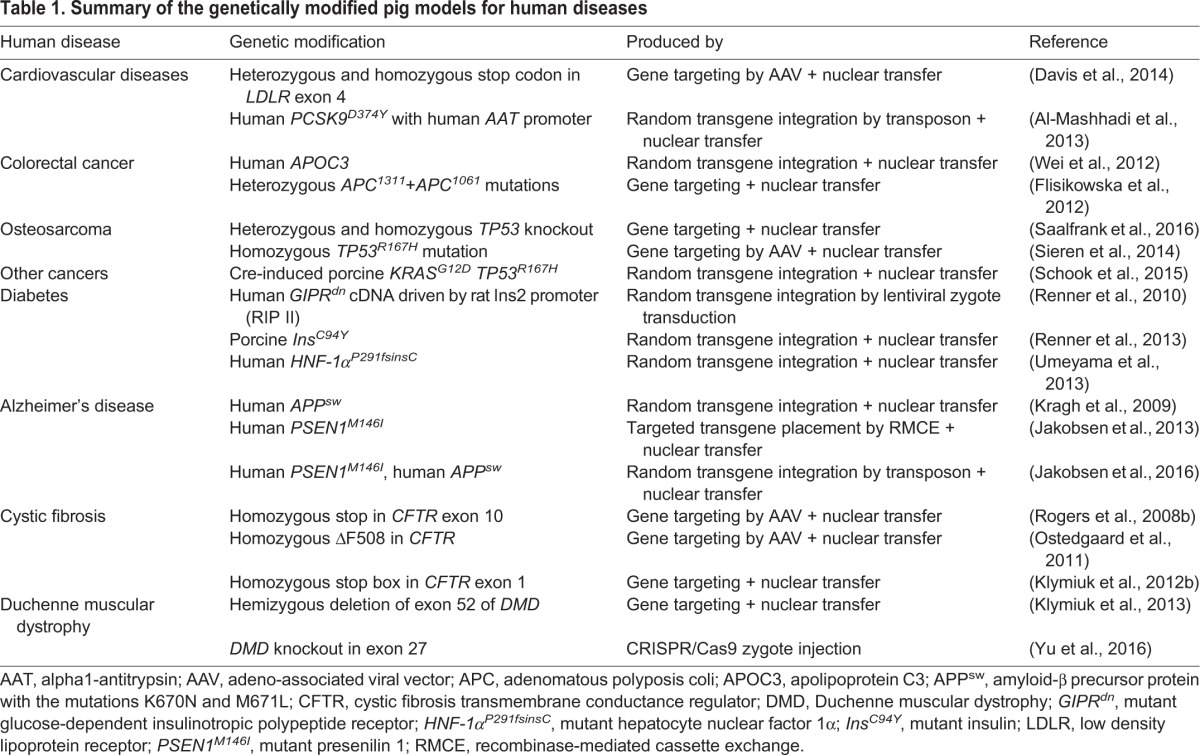
**Summary of the genetically modified pig models for human diseases**

## Methods for generating porcine disease models

**Fig. 1.**
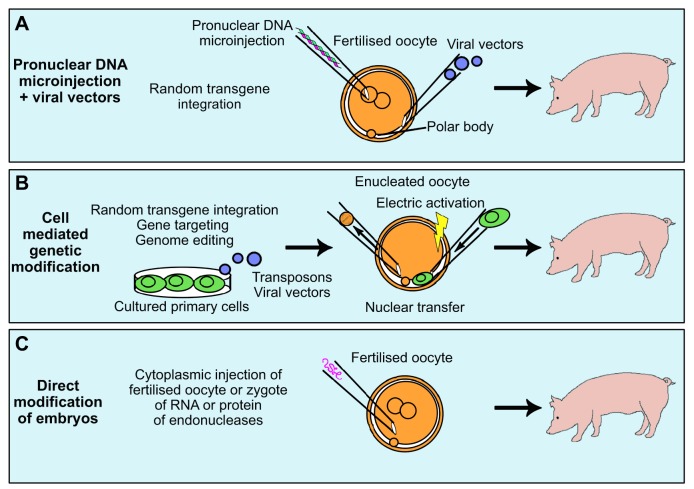
**Methods used to generate genetically modified pigs.** (A) Pronuclear microinjection of DNA results in random integration of transgenes into the genome, but does not enable gene targeted modifications. Viral vectors can also be microinjected to increase the frequency of transgenesis. (B) Somatic primary cells can be cultured and genetically modified by various methods to add random transgenes or for gene targeting. A genetically modified cell (shown in green) is introduced into the perivitelline space of an enucleated oocyte and an electrical pulse used to fuse the cell membranes and simultaneously activate the oocyte. (C) Endonuclease RNA or protein and guide RNA(s) are injected into the cytoplasm of the fertilised oocyte or zygote to directly modify the embryo genome.

**Fig. 2.**
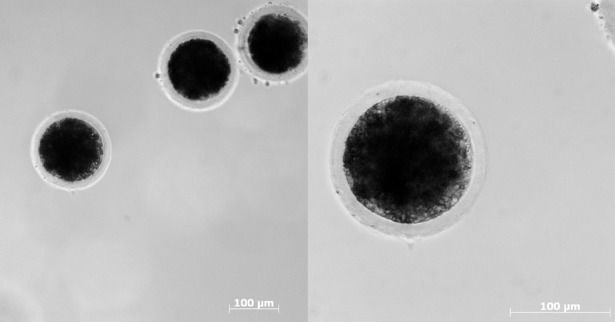
**Porcine oocytes.** Note the opacity of the ooplasm caused by their high lipid content. Porcine oocytes require centrifugation to visualise their pronuclei for microinjection.

Genetic modification of livestock became a reality when transgenic rabbits, sheep and pigs were first produced by pronuclear deoxyribonucleic acid (DNA) microinjection (Fig. 1A) (Hammer et al., 1985). This method is straightforward but inefficient in terms of the proportion of transgenic animals produced, a significant problem with larger species (Hammer et al., 1985; Logan and Martin, 1994; Uchida et al., 2001). Porcine oocytes are also problematic for microinjection as their high lipid content makes them almost opaque and centrifugation is required to visualise the pronuclei (Fig. 2) (Kikuchi et al., 2002a). DNA microinjection in its basic form also results in random transgene integration, which lacks the precision and power of gene targeting. Nevertheless, this remained the only practical technique available to livestock biotechnologists for over a decade (Schnieke et al., 1997).

Meanwhile, the development of gene targeting via homologous recombination (HR) (see Glossary, Box 1) in mouse embryonic stem (ES) cells revolutionised the genetic modification of mice (Evans and Kaufman, 1981; Smithies et al., 1985; Thomas and Capecchi, 1987). The potential of this technology for livestock was recognised at an early stage, but the generation of fully functional ES, embryonic germ, or induced pluripotent stem cells from livestock species that were capable of germline transmission has been unsuccessful (Nowak-Imialek and Niemann, 2012). The search for a functional equivalent led to the development of nuclear transfer from primary somatic cells that could be transfected and analysed in culture. This was used first for random transgenesis (Schnieke et al., 1997) and, subsequently, for gene targeting (McCreath et al., 2000). The method was first demonstrated in sheep but soon extended to pigs (Dai et al., 2002; Lai et al., 2002), where it continues to be a mainstream approach.

**Box 1. Glossary**

**Chylomicron** – lipoprotein particles that are composed of triglycerides, cholesterol, phospholipids and apolipoproteins, including APOE and APOC.

**CRISPR/Cas9** – the CRISPR/Cas9 system consists of the clustered regularly interspaced short palindromic repeat (CRISPR) locus, which contains sequences that guide the endonuclease Cas9 (CRISPR-associated) to foreign DNA via base complementary to cause targeted DNA cleavage.

**Homologous recombination (HR)** – nucleotide sequence exchange between two similar or identical sequences.

**Maturity onset diabetes of the young (MODY)** – a type of diabetes that is associated with monogenetic defects in β-cells and characterised by impaired insulin secretion (insulin function itself remains normal).

**Meconium ileus** – obstruction of the intestine by sticky meconium (the first stool of mammalian infants).

**Nuclear transfer** – transfer of a nucleus into an enucleated oocyte.

**Recombinase-mediated cassette exchange (RMCE)** – a recombination procedure that facilitates recombinases like Cre and Flp to replace, turn or remove gene cassettes.

***ROSA26* locus** – a genomic locus found in mice that yields ubiquitous and constitutive expression of any gene introduced into it via gene targeting. Homologues exist in pig and human and are called the same name.

**Site-specific recombinase** – enzymes that aid site-specific, not random, recombination processes in the cell.

**Transcription activator-like effector nuclease (TALEN)** – a complex consisting of the transcription activator-like effector (TALE), a DNA-binding transcription factor, and the nuclease domain of the FokI restriction enzyme, which creates targeted DNA breaks.

**Zinc finger nuclease (ZFN)** – an artificial construct composed of a DNA-binding zinc finger protein and the nuclease domain of the FokI restriction enzyme, which creates targeted DNA breaks.

Gene targeting in pigs is informed by mouse studies, and aided by bioinformatics and genome sequence data (Groenen et al., 2012). HR in primary porcine somatic cells is, however, much less efficient than in mouse ES cells, although some loci, such as porcine *ROSA26*, support efficient gene targeting (Li et al., 2014). The relatively short lifespan of cultured primary cells also strictly limits the time available for *in vitro* manipulation and cell expansion, while maintaining the ability to generate animals by nuclear transfer (Schnieke et al., 1997). Nuclear transfer is itself difficult and time intensive and, despite steady improvements, produces live viable healthy offspring with relatively low efficiency (Callesen et al., 2014; Kurome et al., 2013). Generating gene targeted pigs has thus been technically challenging, considerably more so than generating gene-targeted mice, as attested by the relatively few such pig lines available.

Nevertheless, other important genetic manipulation techniques developed in mouse ES cells have been extended to pigs (Fig. 1), such as site-specific recombination (see Glossary, Box 1) to control gene expression (Leuchs et al., 2012; Li et al., 2014) and the use of recombinase-mediated cassette exchange (RMCE; see Glossary, Box 1) to induce rearrangements (Clark et al., 2007; Jakobsen et al., 2013). Gene targeting using adeno-associated viral vectors (AAVs) has also been established in pigs (Luo et al., 2011). Improved methods of microinjection, including lentiviral vectors and transposon systems, have also increased rates of transgenesis in pigs (Clark et al., 2007; Garrels et al., 2011; Hofmann et al., 2003; Ivics et al., 2014; Whitelaw et al., 2004).

### Gene editing

The development of synthetic, highly specific endonuclease technologies as tools for ‘gene editing’ has probably had the greatest impact on the genetic modification of pigs. The ability to introduce a single double-strand break (DSB) at a unique predetermined site enables genes to be inactivated by insertion or deletion mutations, introduced via nonhomologous end joining (NHEJ) repair, or by targeted sequence replacement via homology-directed repair with an exogenous homologous DNA fragment. The practicality and simplicity of gene editing has steadily improved in successive generations of endonuclease systems, beginning with zinc finger nucleases (ZFNs; see Glossary, Box 1) (Hauschild et al., 2011; Kwon et al., 2013), then transcription activator-like effector nucleases (TALENs; see Glossary, Box 1) (Carlson et al., 2012) and, most recently, through the use of the CRISPR/Cas9 system (see Glossary, Box 1) (Tan et al., 2013).

Highly efficient gene editing makes it possible to carry out genetic modification directly in zygotes and early-stage embryos and thus avoids nuclear transfer altogether. HR in mouse zygotes is normally very infrequent, <0.1% (Brinster et al., 1989), but the use of ZFNs raises this to 1.7-4.5% (Meyer et al., 2010). ZFN-mediated gene editing in embryos was first demonstrated in a nonrodent species, the rabbit, in 2011 (Flisikowska et al., 2011), and was subsequently extended to pigs (Lillico et al., 2013).

**Fig. 3.**
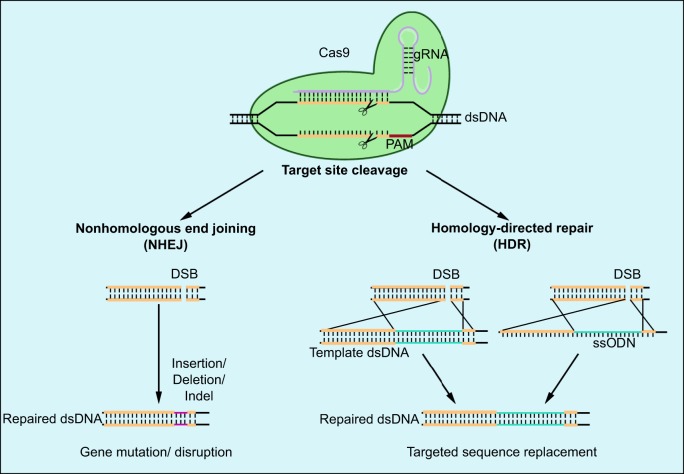
**Gene editing by CRISPR/Cas9 for gene inactivation and targeted sequence replacement.** During gene editing by CRISPR/Cas 9, the endonuclease Cas9 (green) is led by the guide RNA (gRNA) to the genomic target site, where it cleaves the double-stranded DNA (dsDNA) at a point 3-5 bp upstream of the protospacer adjacent motif (PAM). The resulting double-strand break (DSB) can then be repaired by nonhomologous end joining (NHEJ, left) or by homology-directed repair (HDR, right). NHEJ is an error-prone mechanism that can lead to sequence deletion, insertion or both, which can disrupt gene function. The HDR pathway is more precise and uses template DNA to repair the DSB via homologous recombination. The introduction of an exogenous DNA template, as dsDNA or as single-stranded oligodeoxynucleotide (ssODN), allows desired sequence changes to be engineered.

ZFNs and TALENs have now largely been superseded by CRISPR/Cas9, which is equally if not more efficient in inducing DSBs and in stimulating HR (Mali et al., 2013; Wu et al., 2016) (Fig. 3). The CRISPR/Cas9 system also offers improved target specificity, i.e. less off-target activity and better prediction of off-target effects (Cho et al., 2013; Fu et al., 2014; Mali et al., 2013). There have already been reports of potential porcine models of human disease based on gene knockouts generated by the injection of CRISPR/Cas9 components into zygotes (Hai et al., 2014; Wang et al., 2015; Whitworth et al., 2014; Yu et al., 2016). However, gene targeting by HR using CRISPR/Cas9 is more challenging because homology-directed repair is far less frequent than NHEJ, but this approach has been achieved in mice (Chu et al., 2016; Miyaoka et al., 2016). Genetically modified knock-in pigs have also been generated using CRISPR/Cas9 with single-stranded oligodeoxynucleotides as a template with an efficiency of 80% (Zhou et al., 2016).

Gene editing directly in zygotes is likely to replace nuclear transfer as the standard method of generating transgenic pigs because of its efficiency and simplicity.

### Availability of porcine oocytes

An adequate supply of porcine oocytes is essential for nuclear transfer, as are fertilised oocytes or zygotes for newer methods of direct embryo manipulation. While unfertilised and fertilised oocytes can be collected by flushing the reproductive tract, this requires the use of many animals and is cost and labour intensive. Progress in this area came with the development of a robust method of *in vitro* oocyte maturation using ovaries taken from pigs at slaughter, and, more recently, from the production of early porcine embryos *in vitro* (Kikuchi et al., 2002a). This is a multistage process that involves *in vitro* oocyte maturation, *in vitro* sperm preparation, *in vitro* fertilisation (IVF) and embryo culture. Progress is being made in each of these areas, but IVF is at present the limiting step, because of the problem of fertilisation by multiple sperm (Romar et al., 2016). Different groups have reported that freezing semen (Suzuki et al., 2003) or performing IVF in rotating culture (Kitaji et al., 2015) improves monospermy. To date, there have been very few reports of pigs generated from entirely *in vitro*-produced pig embryos (Kikuchi et al., 2002b; Whitworth et al., 2014; Yoshioka et al., 2012).

## Modelling cardiovascular diseases

Cardiovascular diseases (CVDs) are the most common cause of morbidity and death worldwide [World Health Statistics 2017: Monitoring health for the SDGs (http://apps.who.int/iris/bitstream/10665/255336/1/9789241565486-eng.pdf?ua=1)]. Atherosclerosis, the primary culprit, is a chronic inflammatory condition characterised by lipid accumulation, thickening of arterial walls and plaque formation (Lusis, 2000). Chronic expansion of plaques reduces blood flow, and plaque rupture can precipitate acute thrombotic events, such as cardiac infarction and stroke (Falk, 2006).

Mutations of human genes involved in lipoprotein metabolism, including low-density lipoprotein receptor (*LDLR*) and proprotein convertase subtilisin/kexin type 9 (*PCSK9*), can cause familial hypercholesterolaemia (FH) and atherosclerosis or, as in the case of apolipoprotein E (*APOE*), increase disease risk. Several murine CVD models have been generated, including overexpression of wild-type *Pcsk9*, and knockout of *Ldlr* and *Apoe* (Maxwell and Breslow, 2004). Both *Ldlr*- and *Apoe*-deficient mice develop atherosclerotic lesions and hypercholesterolaemia and have provided valuable insights into atherogenic mechanisms (Ishibashi et al., 1993; Plump et al., 1999; Zhang et al., 1992). Importantly, however, these mouse models do not exhibit plaque rupture and thrombosis, a key feature of human atherosclerotic disease (Bentzon and Falk, 2010).

Pigs are well suited to model human CVDs because of similarities in their cardio- and cerebrovascular systems, blood parameters and vessel size, and have been used to develop and improve diagnostic tools and equipment, such as plaque localisation and imaging (Worthley et al., 2000). Also, unlike mice, pigs spontaneously develop atherosclerosis that can be accelerated by an atherogenic diet (Reiser et al., 1959; Skold et al., 1966).

FH has been modelled in Yucatan miniature pigs through AAV-mediated inactivation of *LDLR* (Davis et al., 2014). *LDLR^+/−^* heterozygous pigs developed hypercholesterolaemia, and *LDLR^−/−^* homozygotes developed more severe hypercholesterolaemia and atherosclerotic lesions in coronary arteries and abdominal aorta, disease locations common in humans with CVD. Disease severity was also increased by a diet rich in fat and cholesterol (Davis et al., 2014).

Another porcine FH model has been generated in Yucatan miniature pigs using DNA transposons to introduce a human *PCSK9* transgene that carries the gain-of-function mutation D374Y controlled by a liver-specific human alpha1-antitrypsin promoter (Al-Mashhadi et al., 2013). These pigs showed increased degradation of LDLR, reduced hepatic LDLR, reduced plasma low-density lipoprotein uptake, hypercholesterolemia and atherosclerotic lesions, and have been used to test new imaging techniques to evaluate antiatherosclerotic drugs (Al-Mashhadi et al., 2013). The same model has also been used to investigate the influence of diabetes on atherosclerosis, revealing that poorly controlled blood glucose did not induce more advanced lesions nor increase plaque burden, findings consistent with human studies (Al-Mashhadi et al., 2015). However, none of the pig models described has been reported to show plaque rupture or thrombosis.

Lipid accumulation is an important risk factor in the development of atherosclerotic lesions. Apolipoprotein C3 (APOC3) is a major regulator of plasma triglyceride levels, and its overexpression is closely associated with hypertriglyceridaemia in patients with metabolic syndrome (Cohn et al., 2004). Minipigs that overexpress human *APOC3* show increased plasma triglycerides owing to their delayed clearance, increased very low-density lipoprotein/chylomicron plasma fractions (see Glossary, Box 1) and reduced lipoprotein lipase activity, but not atherosclerotic lesions (Wei et al., 2012).

Thus, while the current generation of pig CVD models do not reproduce all aspects of human CVD pathology, they do recapitulate important early events, including dose-dependent hypercholesterolaemia, hypertriglyceridaemia and atherosclerotic lesions, and thus provide valuable tools to investigate early-stage FH and atherosclerosis, and for the development of new therapies.

## Modelling cancer

Cancers are the second leading cause of death worldwide, and are set to increase as human populations age (World Health Statistics 2017: Monitoring health for the SDGs). In 2012, there were more than 14 million new cancer cases and 8 million cancer-associated deaths (Ferlay et al., 2015). It is thus concerning that only 5% of new anticancer agents are approved for patient use. Reasons for this have been ascribed to the complexity of cancers and the lack of good animal models for preclinical studies (Hutchinson and Kirk, 2011).

Genetically modified mouse strains have been vital tools for cancer research, but have also revealed that mouse and human cancers often differ. For example, murine cells are more easily transformed *in vitro* than human cells (Rangarajan et al., 2004), and different sets of genetic events are required for tumorigenesis (Kendall et al., 2005). Mouse cancer models might not, therefore, always provide the best representation of human disease.

Porcine oncology is a new field and the extent to which pigs replicate human cancers will become clearer as more models are characterised. Adam et al. were the first to address this question, using autologous transplantation of primary porcine cells transformed with viral oncogenic cDNAs (e.g. dominant-negative *Tp53^DD^*, *c-Myc^T58A^* and *H-Ras^G12V^*) to demonstrate that tumorigenesis in pigs resembles that in humans (Adam et al., 2007). Tumorigenesis has also been reported in pigs carrying randomly integrated transgenes that encode Cre-dependent *KRAS^G12D^* and *TP53^R167H^* mutations (Box 2) (Schook et al., 2015). Primary porcine mesenchymal stem cells (MSCs) have also been transformed stepwise into sarcoma cells, showing that they resemble human MSCs in requiring the perturbation of TP53, RB1, KRAS and MYC signalling pathways to become fully transformed (Saalfrank et al., 2016).

**Box 2. Generating inducible oncogenic mutations in pigs**

The controlled activation of inducible and site-specific oncogenic mutations can mimic the spontaneous events that initiate and drive the progression of human cancers. This can be achieved by the introduction of oncogenic mutations silenced by an upstream transcriptional stop signal. Site-specific recombination, e.g. via Cre, excises the stop signal and allows the mutant gene to be expressed. Pigs that express the recombinase in a tissue-specific and/or drug-inducible manner can then be cross bred to activate mutant alleles in chosen tissues (Jin et al., 2014; Klymiuk et al., 2012a; Luo et al., 2014). Local administration of Cre *in vivo* via viral vectors is also possible (Schook et al., 2015).

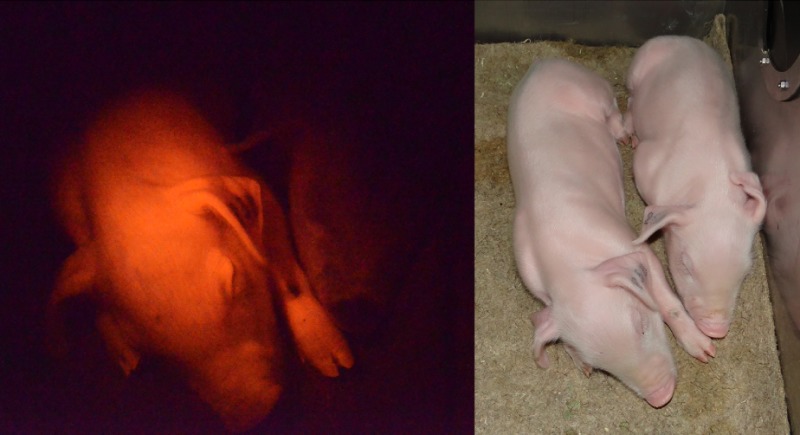

To monitor the pattern and extent of tissue-specific and/or drug-inducible Cre recombination *in vivo*, we have developed a ‘Cre-reporter’ pig that carries a dual fluorochrome cassette that switches expression from red to green after Cre recombination, enabling recombinase activity to be directly visualised (Li et al., 2014) [see figure above showing a piglet that carries a dual fluorochrome cassette in the *ROSA26* locus (left) next to a wild-type piglet (right)]. Inducible porcine models have also been generated using the latent oncogenic mutations *KRAS^G12D^* (Li et al., 2015; Schook et al., 2015) and *TP53^R167H^* (Leuchs et al., 2012; Schook et al., 2015), a mutation that is orthologous to the human R175H *TP53* mutation; both of these represent mutations commonly found in human cancer (Levine and Oren, 2009; Pylayeva-Gupta et al., 2011).

The more recombinase driver lines that become available, the wider the range of tissues and cancers that can be investigated. This is an area where collaboration and synergy between livestock biotechnology researchers is clearly valuable.

### Modelling colorectal cancer

Colorectal cancer (CRC) is the fourth most common cancer worldwide in both sexes (Ferlay et al., 2015). More than 80% of all sporadic cases are initiated by the functional disruption of the tumour suppressor gene adenomatous polyposis coli (*APC*) (Fearnhead et al., 2001; Morán et al., 2010). *APC* mutations are also responsible for a hereditary predisposition to CRC, familial adenomatous polyposis (FAP) (Kinzler et al., 1991). FAP varies considerably in severity, but patients typically develop adenomatous polyps in the colon and rectum in early life and have greatly increased risk of CRC (Croner et al., 2005).

Many murine models, most based on *Apc* mutations, have been generated to model human FAP (Karim and Huso, 2013). However, mutation of *Apc* alone does not replicate the pattern of human polyposis. For example, the widely used *Apc^Min^* mouse develops polyps mainly in the small intestine, and not in the colon, as in FAP patients (Karim and Huso, 2013). More complex mouse models based on additional mutant genes and models that use tissue-specific and locally activated oncogenes have been more successful at modelling FAP (Fearon, 2011; Hung et al., 2010; Tetteh et al., 2016).

Evidence is accumulating that pigs are perhaps more suitable than mice to model CRC. We have generated pigs that carry a translational stop signal at codon 1311 in porcine *APC* (*APC^1311^*), which is orthologous to the human *APC^1309^* mutation responsible for a severe form of FAP. These pigs develop polyps in the colon and rectum as early as 4 months of age, which display epithelial features that are typical of the adenoma-carcinoma sequence, including aberrant crypt foci, and adenomatous polyps with low- and high-grade intraepithelial dysplasia (Flisikowska et al., 2012). Dysplastic adenomas also exhibit loss of APC heterozygosity; a hallmark of human FAP and of sporadic CRC (Albuquerque et al., 2002). Adenomas also showed marked accumulation of β-catenin and high expression of the β-catenin target, c-MYC, and frequent phosphorylation of ERK1/2 (MAPK3/1), a marker of MAPK pathway activation, and a known driver of intestinal tumorigenesis. Although invasive carcinoma has not yet been observed in this APC model, this is probably a function of time. Because mutation of porcine *APC* is sufficient to initiate polyposis without any further engineered mutation, spontaneous events that drive the transition from polyps to cancer can be investigated over time by colonoscopic monitoring and biopsy using standard human equipment. This is not possible in mice and would be very difficult in humans. Comparative transcriptome analysis of high- and low-grade porcine adenomas has also revealed the differential expression of gene sets similar to that in humans, as well as the upregulation of genes known to play a role in human CRC, and interesting new candidate genes involved in precancer development (Flisikowska et al., 2017). Heterozygous *APC* knockout pigs have also been generated by TALEN-mediated inactivation in cultured cells, but so far no phenotypic analysis has been reported (Tan et al., 2013).

The recapitulation of early human CRC pathogenesis was an important step in establishing pigs as a resource for studying human cancers. The FAP model is the first in a series of porcine cancer models to be generated, as discussed further below and in Box 2.

### Modelling osteosarcoma

Osteosarcoma is relatively rare, but is the predominant form of primary bone cancer (Mirabello et al., 2009). It mainly affects young people and is highly malignant, requiring aggressive surgical resection and cytotoxic chemotherapy, the effects of which can be devastating (Durfee et al., 2016). There is thus a pressing need for animal models of this cancer to improve its surgical management, to develop new drugs and to better understand the molecular basis of its initiation and progression. Most human osteosarcomas are sporadic and of unknown cause, but can arise after radiation treatment. They frequently have *TP53* mutations (Overholtzer et al., 2003) and/or alterations that affect cell cycle checkpoints, such as RB1 (Kansara et al., 2014). Patients with Li-Fraumeni syndrome, which is linked to *TP53* mutations, are also predisposed to osteosarcoma (Ognjanovic et al., 2012).

*Trp53* inactivation in mice results in diverse cancers, with ∼25% of heterozygotes and ∼4% of homozygotes developing osteosarcomas; homozygotes mainly develop lymphomas (Jacks et al., 1994). Improved mouse osteosarcoma models have been developed based on conditional *Trp53* inactivation in the osteogenic lineage. These show highly penetrant osteosarcoma formation, but have been criticised because the primary tumours predominantly affect the axial skeleton, in contrast to human osteosarcomas, which tend to arise in the long bones of the limbs (Guijarro et al., 2014).

We have generated pigs that carry a latent *TP53^R167H^* mutation in exon 5 that can be activated by Cre-mediated excision of an upstream transcriptional stop signal (Box 2). In its uninduced form, transcription of the major *TP53* transcript is blocked by a transcriptional stop signal (Leuchs et al., 2012; Saalfrank et al., 2016). Pigs heterozygous for the uninduced allele develop osteosarcomas after 16 months of age, while homozygotes show multiple osteosarcomas at 7-8 months (Leuchs et al., 2012; Saalfrank et al., 2016). The sarcomas primarily affect the long bones, skull and mandible, mirroring human pathology (Guijarro et al., 2014; Saalfrank et al., 2016). Porcine osteosarcoma cells show cytogenetic abnormalities characteristic of human *TP53*-mutant osteosarcoma (Boehm et al., 2000), including abnormal giant nuclei, micronuclei and multinuclear cells with fragmented nuclei and atypical mitotic figures (Saalfrank et al., 2016). Human osteosarcomas show genome-wide DNA instability (Overholtzer et al., 2003), and pig osteosarcoma cells are predominantly karyotypically abnormal (Saalfrank et al., 2016). Also, as in humans, pig osteosarcoma cells show increased resistance to radiation (Saalfrank et al., 2016). Data so far indicate that osteosarcoma is the predominant pathophenotype in these pigs. Little is known about the origin of human osteosarcoma, and the porcine model provides a valuable resource to study the genetic events involved.

Sieren et al. have generated Yucatan minipigs that carry an R176H mutation in the endogenous *TP53* gene but, unlike the model above, this is expressed ubiquitously (Sieren et al., 2014). Heterozygous *TP53^R176H^* mutant pigs showed no tumour development even at 30 months of age, while those homozygotes that reached sexual maturity developed a variety of neoplastic lesions, including osteogenic tumours, lymphomas and renal tumours, recapitulating what has been observed in humans and mice with orthologous mutations.

The difference between the pathophenotypes exhibited by these two models is interesting and comparative analysis could shed light on the events that initiate this deadly disease.

## Modelling diabetes mellitus

Diabetes mellitus is a diverse group of conditions characterised by loss of control of blood glucose levels. By far the most common form is type 2, which is mainly caused by insulin resistance combined with relative insulin deficiency and associated with excess body weight (American Diabetes, 2013). Many rodent models that target insulin signalling and action have provided valuable insights into diabetic disease mechanisms (Aigner et al., 2008; King, 2012).

Following food intake, the incretin hormones glucose-dependent insulinotropic polypeptide (GIP) and glucagon-like peptide-1 (GLP1; also known as GCG) are secreted to enhance glucose-induced insulin secretion (Baggio and Drucker, 2007). GIP function is impaired in type 2 diabetes, suggesting its involvement in early disease pathogenesis (Nauck et al., 2004). To model type 2 diabetes, Renner et al. generated pigs that express a human dominant-negative GIP receptor mutant (*GIPR^dn^*) in pancreatic islets controlled by the rat insulin promoter (Renner et al., 2010). At 11 weeks of age, these pigs showed reduced glucose tolerance due to delayed insulin secretion, and reduced insulin secretion and pancreatic β-cell mass with increasing age. The reduction in β-cell mass is caused by diminished cell proliferation and survival as a result of poor GIP signalling, consistent with findings in other species (Kim et al., 2008). Metabolic studies revealed changes in plasma amino acids and lipids that correlate significantly with β-cell mass (Renner et al., 2012). This pig model has been used to test whether the drug liraglutide, a GLP1 receptor agonist, can compensate for GIP deficiency by increasing GLP1 signalling (Streckel et al., 2015). The drug was found to improve insulin sensitivity, reduce weight gain and food uptake, but did not stop the loss of β-cell mass (Renner et al., 2016). These findings are similar to those from human diabetes patients, while studies in mouse models of diabetes show more disparate results (Shimoda et al., 2011; Tamura et al., 2015).

Mutations in the human insulin (*INS*) gene, e.g. *INS^C96Y^*, can cause permanent neonatal diabetes mellitus (Stoy et al., 2007). Pigs that express a porcine *INS^C94Y^* mutant insulin transgene (orthologous to human *INS^C96Y^*) in β-cells accumulated misfolded insulin in the endoplasmic reticulum and exhibited β-cell apoptosis. At 8 days after birth, *INS^C94Y^* animals developed cataracts and at 4.5 months they showed signs of permanent neonatal diabetes mellitus, including reduced body weight, decreased β-cell mass and reduced fasting insulin levels (Renner et al., 2013). No diabetes-associated pathological changes were detected in the kidney or nervous tissue during 1 year of observation.

Maturity onset diabetes of the young (see Glossary, Box 1) is characterised by impaired insulin secretion with minimal impact on insulin action (American Diabetes, 2013), and is commonly caused by dominant-negative mutations in the gene encoding hepatocyte nuclear factor 1α (*HNF-1α*) (Yamagata et al., 1996). Piglets carrying a dominant-negative human *HNF-1α^P291fsinsC^* mutation developed hyperglycaemia at 2 weeks, and at 19 weeks showed distinct glomerular nodular lesions in the kidneys, a hallmark of diabetic nephropathy, that expanded over the 10-month observation period (Hara et al., 2014; Umeyama et al., 2009, 2013). However, this model lacks several diabetic renal features characteristic of human diabetic nephropathy (Hara et al., 2014).

Diabetes mellitus has a multitude of phenotypic manifestations that are unlikely to be recapitulated in a single animal model. The models mentioned above each show different features that can be investigated further and could lead to the identification of novel therapeutic targets.

## Modelling Alzheimer's disease

Alzheimer's disease (AD) is a multifactorial progressive disease of the brain, characterised by memory loss and disorientation, and accounts for 50-80% of human dementia cases (Winblad et al., 2016). Although its aetiology is not fully understood, it features several key disease hallmarks, including the formation of extracellular beta amyloid protein (Aβ) senile plaques and of intraneuronal neurofibrillary tangles mainly composed of tau protein, as well as neuronal dysfunction and cell death (Ballard et al., 2011). Familial forms of AD are caused by missense gain-of-function mutations in the genes encoding amyloid-β precursor protein (*APP*), presenilin 1 (*PSEN1*) and presenilin 2 (*PSEN2*) (Wu et al., 2012). Abnormal processing and clearance of the transmembrane APP by secretase complexes, in part composed of PSEN1 and PSEN2, lead to Aβ senile plaque development (Citron et al., 1997; De Strooper et al., 1998; Wolfe et al., 1999). Although the aetiology of sporadic AD is more complex than the familial form, the clinical, neuropathological and biochemical similarities have led researchers to replicate the causative genetic lesions in animals.

Transgenic mice that express mutant human *APP* (e.g. double mutant K670N, M671L; V717F) develop senile plaques, but not neurofibrillary tangles or neuronal loss (Games et al., 1995; Hsiao et al., 1996; LaFerla and Green, 2012), and this was also the case when *APP* mutations were combined with *PSEN1* mutations (Takeuchi et al., 2000), indicating that mice are not suitable to model AD.

Kragh et al. have generated Göttingen minipigs that carry a randomly integrated human *APP^sw^* transgene with two mutations (K670N and M671L) driven by the human platelet derived growth factor-beta promoter (Kragh et al., 2009). Despite high transgene expression in the brain, mutant pigs showed no phenotype. The same group also generated minipigs with a human mutant *PSEN1^M146I^* transgene driven by a cytomegalovirus (CMV)-enhanced human UbiC promoter, produced by RMCE and transposon delivery (Jakobsen et al., 2013). Although mutant PSEN1 protein was expressed, there was again no evidence of an AD phenotype. Jakobsen et al. have also generated double transgenic Göttingen minipigs that carry human *PSEN1^M146I^* and *APP^sw^* transgenes. This combination caused an increase in intraneuronal Aβ plaque formation between 10 and 18 months of age (Jakobsen et al., 2016). The authors hypothesised that this might be the first step in AD pathology and a precursor to extracellular senile plaque formation as similar developments have been observed in mice. Further analyses over time are clearly necessary.

These porcine models recapitulate early-stage human AD and confirm previous findings that porcine brain biology is similar to human (Dickerson and Dobbing, 1967; Glauser, 1966; Thibault and Margulies, 1998). As such, they will be useful in studying early-stage AD, but whether a representative model of the full disease can be produced in pigs or another animal remains to be seen.

## Modelling cystic fibrosis

Cystic fibrosis (CF), one of the most common autosomal recessive genetic disorders in populations of northern European extraction, is caused by dysfunction of the CF transmembrane conductance regulator (CFTR), a chloride channel present in the epithelial lining of several tissues, including the airways, intestine, pancreatic ducts, testes and sweat glands (Gadsby et al., 2006). Many *CFTR* mutations have been identified, but 70% of cases are caused by deletion of a phenylalanine at position 508 (ΔF508) (Boyle and De Boeck, 2013; Rogers et al., 2008a). The most serious consequence of impaired chloride channel function caused by *CFTR* mutations is clogging of the airway with mucus, leaving it susceptible to bacterial infections, the main cause of morbidity and mortality (Stoltz et al., 2015). The intestine, pancreas, reproductive tract and biliary system are also affected by this disease.

Several *Cftr* mutant mouse models, including a ΔF508 model, have been generated, but none replicate obstructive lung disease (Wilke et al., 2011). Other species, including sheep and pigs, have been investigated in the search for more representative models. Pigs share similarities in lung function and anatomy with humans, and have been used to model pulmonary abnormalities that play key roles in CF, including infection and inflammation (Pabst, 1996; Pabst and Binns, 1994). *CFTR* mutant pigs have been generated in various ways, for example, by introducing a translational stop codon in exon 10 (Rogers et al., 2008b), the ΔF508 mutation in exon 10 (Ostedgaard et al., 2011), and a stop box in exon 1 (Klymiuk et al., 2012b). All three of these models replicate human pathology equally well. Piglets show features characteristic of CF, including exocrine pancreatic destruction, vas deferens abnormalities, focal biliary cirrhosis, and lung disease marked by inflammation and infection (Ostedgaard et al., 2011; Rogers et al., 2008b; Stoltz et al., 2013). Consistent with findings in humans, CF pigs also show abnormal tracheal structures, as well as axonal and demyelinating neuropathy (Klymiuk et al., 2012b; Meyerholz et al., 2010; Reznikov et al., 2013). However, all piglets also showed a severe form of meconium ileus (see Glossary, Box 1), which is lethal in early life. This occurs in humans but is much less common, affecting 15% of CF patients (Stoltz et al., 2013). Stoltz et al. have thus generated a ‘gut-corrected’ model that incorporates a normal *CFTR* transgene controlled by a *Fabp2* promoter to restore *CFTR* expression in the intestine and thus viability (Stoltz et al., 2013). These animals have been used to test *CFTR* gene therapy using viral vectors, a treatment that successfully improved anion transport, tracheal surface lipid pH and bacterial killing (Cooney et al., 2016; Steines et al., 2016).

The three porcine models generated to model CF are all well characterised and show accurate replication of the human pathology. They are probably the most advanced of the porcine disease models and are already being used to test *CFTR* gene therapy.

## Modelling Duchenne muscular dystrophy

Duchenne muscular dystrophy (DMD) is an X-linked lethal disorder characterised by progressive muscle weakness and wasting that affects approximately 1 in 3500-5000 human males (Mendell et al., 2012). It is caused by frameshift mutations in the *DMD* gene, which lead to loss of the muscle protein dystrophin. Mutation hotspots have been identified in exons 3-7 and 45-55 of the *DMD* gene (Koenig et al., 1989).

Several mouse models of DMD have been generated but, unlike humans, mice require more than one mutation to replicate the human disease phenotype (Araki et al., 1997; McGreevy et al., 2015). Spontaneous *DMD* mutations have also been identified in cats and dogs (McGreevy et al., 2015; Nakamura and Takeda, 2011). Dog models replicate the human phenotype better than do mouse models of this disease, but the causative mutation in dogs does not occur in human DMD patients (Yu et al., 2015).

Male pigs that carry a *DMD* gene lacking exon 52 replicate signs of human DMD pathology, including loss of dystrophin in skeletal muscle, progressive muscular dystrophy, increased serum creatine kinase levels and impaired mobility (Klymiuk et al., 2013). However, the animals’ lifespan is reduced to ≤3 months, precluding natural breeding. Transcriptome analysis of skeletal muscle at 3 months resembled that of human DMD patients (Klymiuk et al., 2013), as did proteome analysis (Frohlich et al., 2016). The mutation used occurs commonly in human DMD and can be treated by exon 51 skipping, as shown in human patients (Goemans et al., 2011; Heemskerk et al., 2010). This model could be useful in devising treatment strategies for DMD, but its practical value would be greatly enhanced by improved viability.

Another porcine DMD model has been generated by zygotic injection of Cas9 messenger ribonucleic acid (mRNA) and a single guide RNA (sgRNA) that targets *DMD* at exon 27 (Yu et al., 2016). Analysis of the mosaic founder showed 70% and 60% of dystrophin alleles to be mutated in skeletal and smooth muscle, respectively. Although mutations in exon 27 are not found in humans, piglets carrying this deletion replicate the degeneration and disorganisation of cardiac and skeletal muscle seen in human patients, and also reduced thickness of intestine and stomach smooth muscle. However both founder animals died of unreported causes. The group tested 14 likely CRISPR/Cas9 off-target sites, but could not detect such activity. Noteworthy is the extremely high efficiency (50%) of *DMD* targeting in zygotes, which if repeatable means that further animals and a wider range of mutations should be easy to generate.

## Future perspectives

Niels Bohr reportedly joked that ‘Prediction is difficult, especially about the future’. This is a valuable caution given recent rapid changes. Nevertheless, here we indicate those techniques that are likely to help generate porcine models of human disease and the technical issues that need to be solved in the coming years.

Multiple recombinase systems using Flp, Cre and Dre recombinases or PhiC31 integrase are very powerful tools in mice that can be used to effect sequential genetic modifications and to control the expression and inactivation of multiple genes (Schönhuber et al., 2014). Extension of such methods to pigs would be very useful, enabling, for example, the roles of particular genes in cancer pathogenesis to be studied in different tissues and over time. These techniques also allow the ablation of specific cell types to investigate their role in a disease process (Schönhuber et al., 2014).

While our discussion of CRISPR/Cas9 has concentrated on germline alterations, this system also provides a means of inducing precise somatic mutations *in vivo*. Local gene editing within a tumour entity could, for example, be used to replicate the accumulation of somatic mutations responsible for cancer progression. The larger body size of pigs offers an advantage over rodents, allowing access and delivery of CRISPR/Cas9 using standard human surgical and endoscopic methods. Delivery of Cas9 via size-limited AAVs will be aided significantly by the recently developed intein-mediated split-Cas9, and by the discovery of a smaller Cas9 orthologue from *Campylobacter jejuni* (Kim et al., 2017; Truong et al., 2015). The repertoire of gene editing tools and variants suited to particular tasks will no doubt increase and improve the refinement with which diseases can be modelled and studied.

Nuclear transfer is still the standard method of producing gene-targeted pigs, but the difficulty and inefficiency of the process pose serious limitations. Direct modification of early embryos is currently the most promising alternative, and ever more sophisticated modifications are likely to be possible. This, however, places the focus on the need for reliable *in vitro* production of pig embryos, and in particular on solving the long-standing problem of polyspermy with porcine IVF. A quite different means of modifying the mammalian germline was first demonstrated in rats more than two decades ago. Spermatogonial stem cells (SSCs) can be transferred between animals and produce viable sperm in the recipient (Brinster and Avarbock, 1994; Brinster and Zimmermann, 1994), opening the possibility of modifying SSCs *in vitro* then transferring them to recipient ‘founder’ males that transmit the modified genotype via their sperm, with no need for embryo manipulation.

Excitingly, this technology has recently been extended to pigs by Park et al. (2017), who demonstrated transplantation of SSCs into boars rendered germline deficient by homozygous knockout of *NANOS2*. This method does, however, require the development of conditions for long-term SSC culture and transfection *in vitro*. These methods are not yet available for pig, but have been established in mice (Kubota et al., 2004).

Leaving technology aside, there are also whole new areas of biomedicine yet to explore. For example, human viral diseases, such as hepatitis B and human immunodeficiency virus (HIV), have been modelled in mice through the introduction of transgenic viral receptors (Chisari and Oldstone, 1996; Hatziioannou and Evans, 2012; Koike et al., 1994; Yang et al., 2014), but these diseases have yet to be investigated in pigs. Another area with great potential is the study of pigs with a humanised immune system. Mice have been humanised by genetic ablation of lymphocytes and re-colonisation with human immune cells (Ito et al., 2014; Suzuki et al., 2016). Pigs engineered in this way could provide information about the response of the human immune system to cancers, infections and grafts, and also provide an orthotopic tumour xenograft model to study the therapeutic response of a patient's tumour, as demonstrated in mice (Hidalgo et al., 2014). Finally, somewhat controversial, but of potential biomedical value, are chimeric pigs that carry human organs (Wu et al., 2017). With proper safeguards and ethical approval, pigs carrying human organs, such as the liver, could provide a powerful preclinical tool to study drug pharmacokinetics and toxicity and could also provide a possible source of organs for transplantation.
